# IL‐1α cleavage by inflammatory caspases of the noncanonical inflammasome controls the senescence‐associated secretory phenotype

**DOI:** 10.1111/acel.12946

**Published:** 2019-03-27

**Authors:** Kimberley A. Wiggins, Aled J. Parry, Liam D. Cassidy, Melanie Humphry, Steve J. Webster, Jane C. Goodall, Masashi Narita, Murray C. H. Clarke

**Affiliations:** ^1^ Division of Cardiovascular Medicine Department of Medicine University of Cambridge Cambridge UK; ^2^ Cancer Research UK Cambridge Institute, University of Cambridge Cambridge UK; ^3^ Division of Rheumatology Department of Medicine University of Cambridge Cambridge UK; ^4^ Present address: Department of Veterinary Medicine University of Cambridge Cambridge UK

**Keywords:** caspase, IL‐1, IL‐1 alpha, inflammasome, inflammation, senescence, senescence‐associated secretory phenotype

## Abstract

Interleukin‐1 alpha (IL‐1α) is a powerful cytokine that modulates immunity, and requires canonical cleavage by calpain for full activity. Mature IL‐1α is produced after inflammasome activation and during cell senescence, but the protease cleaving IL‐1α in these contexts is unknown. We show IL‐1α is activated by caspase‐5 or caspase‐11 cleavage at a conserved site. Caspase‐5 drives cleaved IL‐1α release after human macrophage inflammasome activation, while IL‐1α secretion from murine macrophages only requires caspase‐11, with IL‐1β release needing caspase‐11 and caspase‐1. Importantly, senescent human cells require caspase‐5 for the IL‐1α‐dependent senescence‐associated secretory phenotype (SASP) in vitro, while senescent mouse hepatocytes need caspase‐11 for the SASP‐driven immune surveillance of senescent cells in vivo. Together, we identify IL‐1α as a novel substrate of noncanonical inflammatory caspases and finally provide a mechanism for how IL‐1α is activated during senescence. Thus, targeting caspase‐5 may reduce inflammation and limit the deleterious effects of accumulated senescent cells during disease and Aging.

## 1 INTRODUCTION

Inflammation has evolved to protect the host from acute insults such as infection or physical injury. However, chronic inflammation is associated with many age‐related diseases such as atherosclerosis, osteoarthritis and cancer. Senescent cells that drive inflammation also play pivotal roles in these diseases (Childs et al., 2016; Jeon et al., 2017; Kang et al., 2011), and naturally accumulate in tissues during ageing (Baker et al., 2016). Strikingly, removal of senescent cells can prevent the development of disease and also reverse natural features of ageing (Bakeretal., 2016, 2011; Childs et al., 2016; Jeon et al., 2017; Kang et al., 2011). Thus, understanding how senescence drives inflammation is of critical importance in health, disease and ageing.

Senescence is a protective mechanism that induces permanent cell cycle arrest to prevent transmission of defects to the next generation, particularly to stop malignant transformation. Replicative senescence occurs after repeated cell division critically shortens telomeres, while induced senescence occurs after oncogene activation, mitochondrial deterioration, oxidative stress or DNA damage (Munoz‐Espin & Serrano, 2014). As ageing drives tumorigenesis and telomere shortening, it induces senescence via both pathways. Most senescent cells develop altered secretory activities known as a senescence‐associated secretory phenotype (SASP). The SASP releases proinflammatory cytokines (e.g., IL‐1, IL‐6) and chemokines (e.g., IL‐8, GROα), growth factors (e.g., G‐CSF, bFGF), and proteases (e.g., MMPs, PAI‐1) (Coppe et al., 2008), conferring diverse activities. Thus, although cell cycle arrest during senescence limits cancer and the SASP instructs clearance of preneoplastic cells (Kang et al., 2011), this is balanced against establishment of a chronic inflammatory microenvironment that can damage tissue, drive disease and promote tumorigenesis if senescent cells persist (Grivennikov, Greten, & Karin, 2010). IL‐1α acts in an autocrine/paracrine fashion to drive the SASP (Gardner, Humphry, Bennett, & Clarke, 2015; Orjalo, Bhaumik, Gengler, Scott, & Campisi, 2009), with upstream expression controlled in part by ATM/ATR liberation of GATA4 from p62‐directed autophagy (Kang et al., 2015) and/or an mTORC1‐dependent pathway (Laberge et al., 2015). However, how IL‐1α is cleaved, activated or released during senescence to enable it to drive the SASP is unknown.

Interleukin‐1 (IL‐1) is an ancient cytokine that exerts effect on both innate and adaptive immunities. IL‐1α and IL‐1β are the principal ligands that bind to the type 1 IL‐1 receptor (IL‐1R1), causing recruitment of the IL‐1 receptor accessory protein (IL‐1RAP) and subsequent interaction with the signalling adapter MyD88 (Dinarello, 2009). A consequent phospho‐signalling cascade activates NF‐κB leading to multiple effects on immunity including cytokine secretion, upregulation of adhesion and/or MHC/costimulatory molecules, increased vascular permeability, TH17 cell differentiation, and effector T‐cell proliferation in the presence of regulatory T cells (Sims & Smith, 2010). These powerful actions of IL‐1 are countered by a receptor antagonist (IL‐1RA), a decoy receptor (IL‐1R2), and production of IL‐1α and IL‐1β as pro‐proteins that require cleavage for full biological activity. IL‐1α is canonically cleaved by calpain, which occurs upon necrosis in some cell types and significantly increases activity (Burzynski, Humphry, Bennett, & Clarke, 2015; Zheng, Humphry, Maguire, Bennett, & Clarke, 2013), while IL‐1β is activated by caspase‐1 (Black et al., 1988) after inflammasome engagement (Martinon, Burns, & Tschopp, 2002). How IL‐1α is released without necrosis is unknown and puzzling since IL‐1α release is inhibited in *Casp1^−/−^*mice (Kuida et al., 1995; Li et al., 1995), even though IL‐1α is not a caspase‐1 substrate. However, the original *Casp1^−/−^*mice have an inactivating passenger mutation in *Casp11 *(Kayagaki et al., 2011), suggesting caspase‐11 might activate IL‐1α. Murine caspase‐11 and the human orthologues caspase‐4 and caspase‐5 are required for noncanonical inflammasome activation in response to intracellular LPS (icLPS) from bacterial infection (Kayagaki et al., 2011; Shi et al., 2014), and control monocyte IL‐1 release (Vigano et al., 2015). Direct binding of LPS to caspase‐11, caspase‐4 or caspase‐5 leads to pyroptosis and/or NLRP3 inflammasome activation, with IL‐1β and/or IL‐1α release (Kayagaki et al., 2011; Shi et al., 2014). However, whether caspase‐4/5 or caspase‐11 requires caspase‐1 to mediate IL‐1α activation, if IL‐1α is only passively released, or if caspase‐4/5 or caspase‐11 can directly cleave and activate IL‐1α in any of these systems is unknown.

We show that IL‐1α is specifically cleaved and activated at a conserved site by caspase‐5 and caspase‐11, but not caspase‐4. Knockdown of caspase‐5 or expression of a caspase site mutant reduces release of IL‐1α after icLPS stimulation. IL‐1α cleavage and release from murine macrophages after icLPS require only caspase‐11, while IL‐1β needs both caspase‐11 and caspase‐1. Importantly, we show caspase‐5 and caspase‐11 are required for senescent cells to establish the IL‐1α‐dependent SASP. Thus, IL‐1α is a direct substrate for inflammatory caspases during noncanonical inflammasome activation and senescence.

## 2 RESULTS

### 2.1 Caspase‐5 cleavage of human IL‐1α at a conserved site increases activity

**Figure 1:**
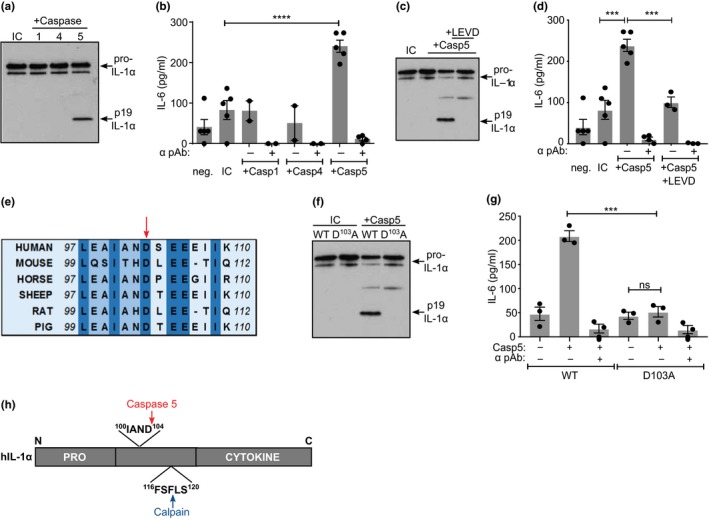
Caspase‐5 cleavage of human IL‐1α at a conserved site increases activity. (a) Western blot for IL‐1α after incubation of pro‐IL‐1α with active caspases, or alone (incubation control; IC). (b) IL‐1‐dependent IL‐6 production by HeLa cells treated with reaction products from pro‐IL‐1α incubated ± active caspases, ±neutralizing IL‐1α antibody (α pAb). (c, d) Western blot (c) and bioactivity (d) of IL‐1α after incubation ± caspase‐5, ±caspase inhibitor LEVD. (e) Multispecies IL‐1α protein alignment showing conserved aspartic acid residue (arrow). (f) Western blot for wild‐type (WT) or mutant D^103^A pro‐IL‐1α after incubation ± caspase‐5, or alone (IC). (g) IL‐1‐dependent IL‐6 production by HeLa cells treated with reaction products from WT or mutant pro‐IL‐1α incubated ± caspase‐5, ±neutralizing IL‐1α antibody (α pAb). (h) Pictograph showing position of cleavage sites in IL‐1α. Data represent mean ± *SEM* of *n* = 3 (g), *n* = 4 (b, d); *p* = **≤0.01, ***≤0.001, ****≤0.0001; ns = not significant

Although noncanonical inflammasomes that utilize caspase‐4 and caspase‐5 result in IL‐1α and/or IL‐1β release (Kayagaki et al., 2011; Shi et al., 2014), their role in cleavage or secretion of IL‐1α is unknown. Thus, we investigated whether IL‐1α could be proteolytically activated by these caspases. Incubation of recombinant pro‐IL‐1α with active inflammatory caspases revealed that caspase‐5 cleaved IL‐1α to a ~19 kDa fragment, while caspase‐1 or caspase‐4 did not (Figure 1a), resulting in increased IL‐1α‐specific cytokine activity (Figure 1b). Both cleavage and increased IL‐1α activities were dependent on caspase‐5 proteolytic activity, as the inhibitor LEVD‐fmk reduced both (Figure 1c,d). Caspases require Asp before the cleavage site, and thus, important substrates show Asp conservation between species. Aligned IL‐1α protein sequences revealed a conserved IAND tetrapeptide motif (Figure 1e), a known target of granzyme B (Afonina et al., 2011), with the Asp present in 81% of all sequences available (Ensembl). Cleavage at D^103^ would produce a ~19 kDa C‐terminal fragment, which is congruent with the size seen by Western blot (Figure 1a). Mutation of Asp^103^ to Ala abolished caspase‐5 cleavage of pro‐IL‐1α (Figure 1f) and thus prevented activation (Figure 1g). This shows that caspase‐5 increases IL‐1α activity by direct processing at a conserved site located adjacent to the calpain site (Figure 1h).

### 2.2 Release of cleaved IL‐1α from human cells requires caspase‐5

**Figure 2:**
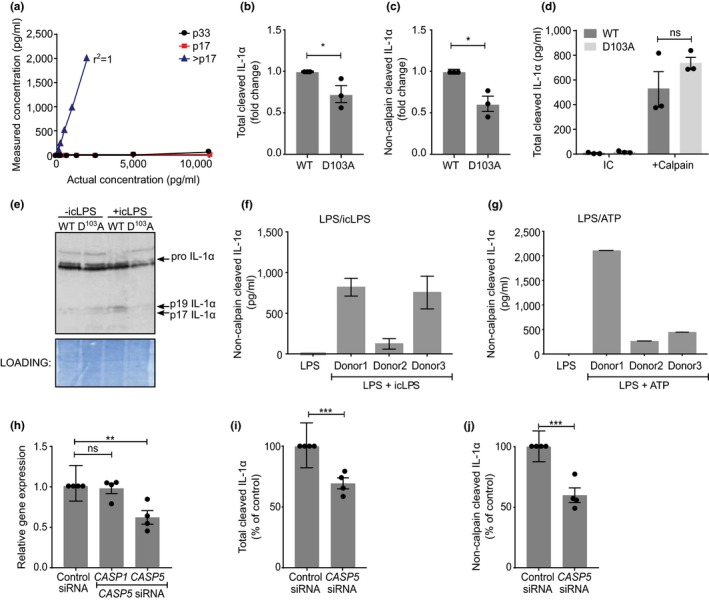
Release of cleaved IL‐1α from human cells requires caspase‐5. (a–d) ELISA data showing detection of recombinant pro‐ (p33), calpain‐cleaved (p17) or non‐calpain‐cleaved (>p17) IL‐1α (a), the level of total cleaved IL‐1α (b) or non‐calpain‐cleaved IL‐1α (c) in the conditioned media of HeLa cells transfected with WT or D^103^A pro‐IL‐1α and treated with intracellular LPS (icLPS), or total cleavage of WT or D^103^A pro‐IL‐1α by calpain (d). (e) Western blot for IL‐1α in HeLa cells transfected with WT or D^103^A pro‐IL‐1α, ±icLPS treatment. (f, g) ELISA data showing level of non‐calpain‐cleaved IL‐1α in the conditioned media of LPS primed human monocyte‐derived macrophages (hMDMs) after icLPS (f) or ATP (g) treatment. (h) qPCR data showing relative gene expression in hMDMs after transfection of control‐ or *CASP5*‐targeted siRNA. (i, j) ELISA data showing level of total cleaved IL‐1α (i) or non‐calpain‐cleaved IL‐1α (j) in the conditioned media of icLPS‐treated hMDMs after transfection of control‐ or *CASP5*‐targeted siRNA. Data represent mean ± *SEM* of *n* = 3 (b–d), *n* = 4 (h–j), or mean ± *SD* of *n* = 3 separate donors (f, g); *p* = *≤0.05, **≤0.01, ***≤0.001; ns = not significant, nd = not detected

Due to the similar size of calpain‐ and caspase‐5‐cleaved IL‐1α, we produced a peptide antibody that was reactive to the region between the calpain and caspase‐5 sites (Supporting information Figure S1a) and developed an ELISA that recognized caspase‐5‐cleaved IL‐1α, but not pro‐IL‐1α or calpain‐cleaved IL‐1α (Figure 2a and Supporting information Figure S2b). We also established an IL‐1α ELISA that recognized total cleaved IL‐1α, but not pro‐IL‐1α (Supporting information Figure S1c). To test IL‐1α processing and release in cells, we expressed pro‐caspase‐5 and either wild‐type (WT) or D^103^A mutant pro‐IL‐1α in HeLa cells, and transfected LPS into the cytosol (icLPS) to activate the noncanonical inflammasome. D^103^A transfected cells released less total cleaved (Figure 2b) and less non‐calpain‐cleaved IL‐1α (Figure 2c). Calpain cleaved D^103^A equivalently to WT pro‐IL‐1α (Figure 2d), showing the mutation only prevents noncanonical processing. Furthermore, HeLa cells expressing WT pro‐IL‐1α produced a 19 kDa IL‐1α fragment that was absent in D^103^A expressing cells after icLPS (Figure 2e). Primary human monocyte‐derived macrophages (hMDMs) primed and then noncanonically activated with icLPS released non‐calpain‐cleaved IL‐1α (Figure 2f). Surprisingly, LPS/ATP‐activated hMDMs also produced non‐calpain‐cleaved IL‐1α (Figure 2g), suggesting caspase‐5 may also cleave some pro‐IL‐1α after canonical stimulation, likely due to the late activation of caspase‐11 by caspase‐1 (Kayagaki et al., 2011). Importantly, knockdown of caspase‐5 in hMDMs (Figure 2h) resulted in decreased release of total cleaved (Figure 2i) and non‐calpain‐cleaved IL‐1α (Figure 2j) after icLPS treatment. This suggests that caspase‐5 can directly process IL‐1α in cells, which results in increased activity and its subsequent release.

### 2.3 Release of cleaved IL‐1α from murine macrophages requires caspase‐11

**Figure 3:**
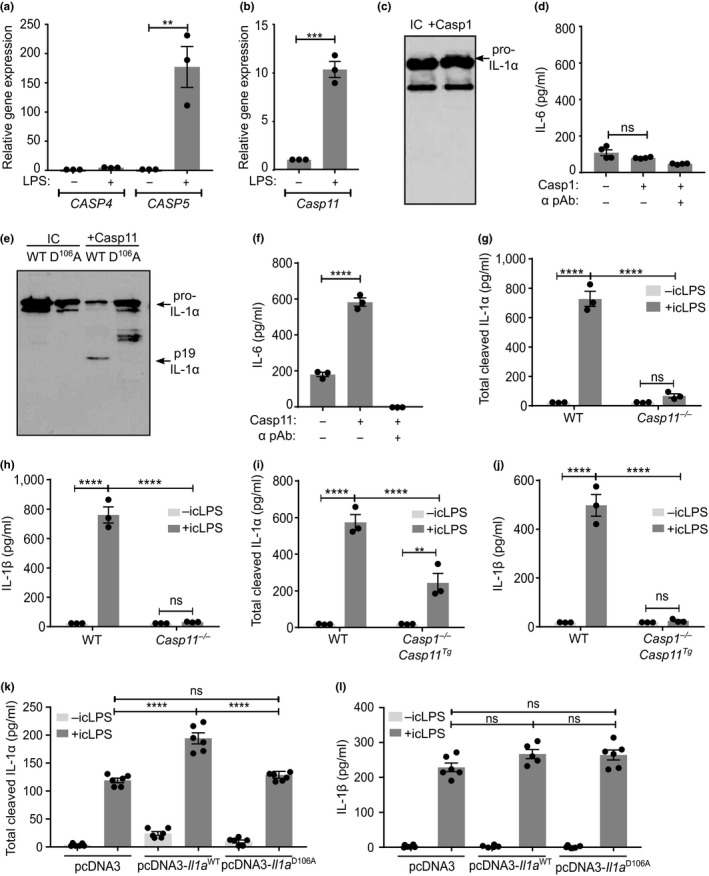
Release of cleaved IL‐1α from murine macrophages requires caspase‐11. (a, b) qPCR data showing changes in *CASP4*, *CASP5 *(a) or *Casp11 *(b) transcript after LPS treatment of primary human (a) or mouse (b) macrophages. (c) Western blot for IL‐1α after incubation of murine pro‐IL‐1α with murine caspase‐1, or alone (IC). (d) IL‐1‐dependent IL‐6 production by murine fibroblasts treated with reaction products from murine pro‐IL‐1α incubated ± murine caspase‐1. (e, f) Western blot of murine wild‐type (WT) or D^106^A pro‐IL‐1α (e) and bioactivity of murine WT pro‐IL‐1α (f) after incubation ± caspase‐11. (g–l) ELISA data showing the level of cleaved IL‐1α (g, i, k) or IL‐1β (h, j, l) detected in the conditioned media of mouse bone marrow‐derived macrophages (mBMDMs) from WT, *Casp11^−/−^*(g, h) or *Casp1^−/−^*/*Casp11* ^Tg ^(i, j) mice, or immortalized mBMDMs transfected with empty, WT *Il1a *or D106A *Il1a *vectors (k, l), followed by LPS priming and then transfection of intracellular LPS (icLPS). Data represent mean ± *SEM* of *n* = 3 (a, b, f–l), *n* = 4 (d); *p* = **≤0.01, ***≤0.001, ****≤0.0001; ns = not significant

Caspase‐11 is the murine orthologue of human caspase‐4 and caspase‐5, with all directly binding icLPS (Kayagaki et al., 2013; Shi et al., 2014), signifying functional conservation. Although caspase‐4 is constitutively expressed (Kajiwara et al., 2014), only caspase‐5 (Figure 3a) and caspase‐11 (Figure 3b) are upregulated by LPS, suggesting greater functional equivalence between caspase‐5 and caspase‐11. Murine caspase‐1 did not process (Figure 3c) or activate (Figure 3d) murine IL‐1α, while IL‐1β was cleaved and activated (Supporting information Figure S2a,b), confirming murine IL‐1α is not a caspase‐1 substrate. However, caspase‐11 cleaved murine pro‐IL‐1α at the conserved D^106^ site (Figure 3e), again increasing activity (Figure 3f). Utilizing bone marrow‐derived macrophages (BMDMs) from WT, *Casp11^−/−^* and *Casp1^−/−^*/*Casp11 *transgenic (Tg) mice showed total loss of cleaved IL‐1α (Figure 3g) and IL‐1β (Figure 3h) secretion by *Casp11^−/−^*BMDMs, as expected (Kayagaki et al., 2011). Importantly, release of cleaved IL‐1α was reinstated in *Casp1^−/−^*/*Casp11* ^Tg^ BMDMs (Figure 3i), suggesting IL‐1α only requires caspase‐11 for cleavage and release after icLPS. The reason *Casp1^−/−^*/*Casp11* ^Tg^ BMDMs do not release WT levels of IL‐1α is thought to be due to incomplete complementation of endogenous *Casp11 *by the *Casp11 *transgene*, *as shown previously (Kayagaki et al., 2011). Interestingly, *Casp1^−/−^*/*Casp11* ^Tg^ BMDMs did not release IL‐1β (Figure 3j), showing IL‐1β requires both caspase‐1 and caspase‐11. To determine whether caspase‐11 processing of pro‐IL‐1α drives IL‐1α release, as opposed to passive leakage after caspase‐11‐induced cell lysis, we expressed low levels of WT or D^106^A murine pro‐IL‐1α in immortalized mBMDMs, and treated with icLPS. Expression of WT IL‐1α significantly increased release of cleaved IL‐1α, while D^106^A mutant expressing macrophages released the same amount of cleaved IL‐1α as empty vector‐transfected cells (Figure 3k). Importantly, no difference in viability (Supporting information Figure S3a,b) or IL‐1β release after icLPS was seen between groups (Figure 3l), indicating effect on IL‐1α only. Together, these data show that caspase‐11 cleavage of murine IL‐1α at D^106^A is required for full activity and release from murine macrophages, while IL‐1β activation and release still require caspase‐1 and caspase‐11.

### 2.4 Caspase‐5 is required for the IL‐1α‐dependent senescent‐associated secretory phenotype

**Figure 4:**
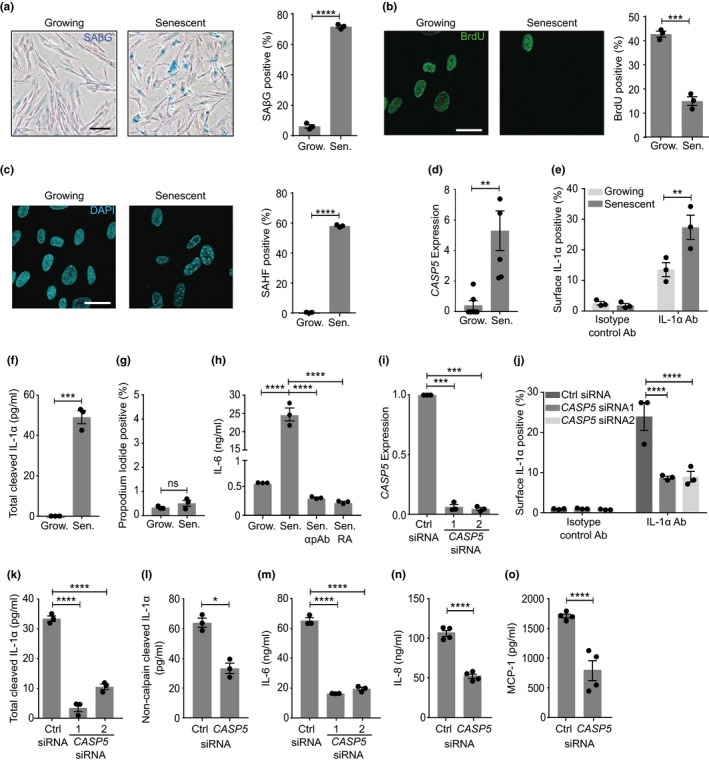
Caspase‐5 is required for the IL‐1α‐dependent SASP. (a–c) Representative images and quantification of senescence‐associated beta‐galactosidase (SAβG) (a), proliferation by BrdU (b) and senescence‐associated heterochromatic foci (SAHF) (c) in growing (Grow.) and senescent (Sen.) IMR‐90 cells (n.b. b, c are the same field of view). (d–g) Caspase‐5 expression by RNA‐Seq (d), cell surface IL‐1α by flow cytometry (e), cleaved IL‐1α in the conditioned media by ELISA (f) and level of cell death (g) in growing and senescent IMR‐90 cells. (h) ELISA data showing the level of IL‐6, ±neutralizing IL‐1α antibody (α pAb) or IL‐1RA (RA) in the conditioned media of growing and senescent IMR‐90 cells. (i) qPCR data showing relative expression of *CASP5 *in senescent IMR‐90 cells after transfection of control‐ (Ctrl) or *CASP5*‐targeted siRNAs. (j–o) Cell surface IL‐1α by flow cytometry (j), or total cleaved IL‐1α (k), non‐calpain‐cleaved IL‐1α (l), IL‐6 (m), IL‐8 (n) and MCP‐1 (o) by ELISA in the conditioned media of senescent IMR‐90 cells after transfection of control‐ or *CASP5*‐targeted siRNA. Data represent mean ± *SEM* of *n* = 3, *n* = 4 (n, o), *n* = 6 (d); *p* = *≤0.05, **≤0.01, ***≤0.001, ****≤0.0001; ns = not significant. Scale bars = 100µm (a), 15 µm (b, c)

The SASP directs clearance of senescent cells, but also drives chronic inflammation that can promote disease and unhealthy ageing. As IL‐1α drives the SASP, we investigated whether caspase‐5 is required for IL‐1α release during senescence. We utilized the well‐characterized tamoxifen‐inducible HRAS^G12V^‐induced senescence of human IMR‐90 fibroblasts. Seven days after tamoxifen, cultures were positive for senescence‐associated beta‐galactosidase (SAβG) (Figure 4a), had reduced proliferation (Figure 4b) and showed senescence‐associated heterochromatic foci (SAHF) (Figure 4c). Senescent cultures showed increased expression of *CASP5 *(Figure 4d), cell surface IL‐1α (Figure 4e and Supporting information Figure S4) and total cleaved IL‐1α in the conditioned media (Figure 4f), compared to growing cells. Importantly, similar low levels of death were found in growing and senescent cultures (Figure 4g), excluding passive release of IL‐1α after cell lysis. Senescent cells released the common SASP cytokines IL‐6 (Figure 4h), IL‐8 and MCP‐1 (Supporting information Figure S5), which were all dependent on IL‐1α. Consistent with this, although pro‐IL‐1β was upregulated in senescent cells, negligible amounts were cleaved to the mature form and none was released from the cell (Supporting information Figure S6a,b). siRNA‐mediated knockdown of *CASP5 *(Figure 4i) led to significantly less cell surface IL‐1α (Figure 4j and Supporting information Figure S4), reduced release of total (Figure 4k) and non‐calpain‐cleaved (Figure 4l) IL‐1α, and a subsequent reduction in IL‐6, IL‐8 and MCP‐1 (Figure 4m–o). Caspase‐5 was also required for the SASP in WI‐38 cells (Supporting information Figure S7a–e). With either cell type, *CASP5 *knockdown did not alter SAβG or proliferation levels (Supporting information Figure S8a,b). The SASP is dependent on cGAS sensing of cytosolic chromatin, which drives interferon signalling (Gluck et al., 2017; Yang, Wang, Ren, Chen, & Chen, 2017). As *CASP5 *is interferon responsive, we tested whether its expression was cGAS‐dependent. *cGAS *knockdown resulted in reduced release of IL‐1α and IL‐6, as expected, but also decreased *CASP5 *expression (Supporting information Figure S9a–d). Together, this suggests that release of cleaved fully active IL‐1α and expression of the subsequent SASP is dependent on caspase‐5, with upstream cGAS potentially controlling *CASP5 *expression.

### 2.5 Caspase‐11 is required for immune surveillance of senescent cells in vivo

**Figure 5:**
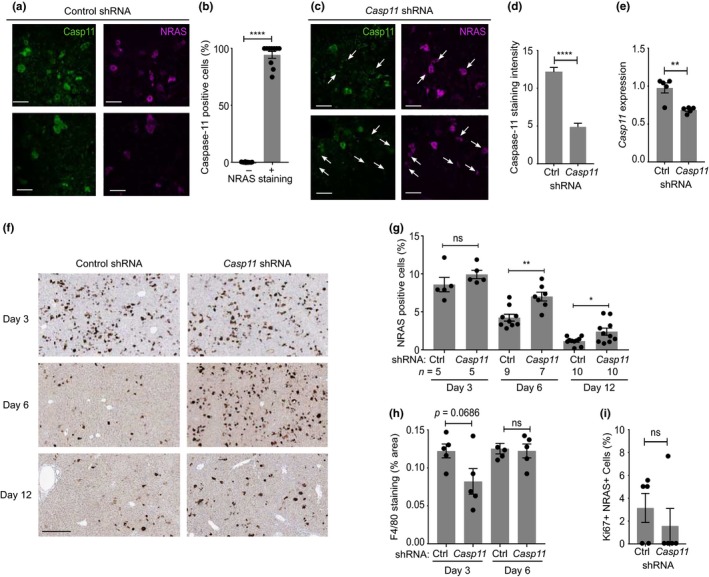
Caspase‐11 is required for immune surveillance of senescent cells in vivo. (a–d) Representative images (a, c) and quantification (b, d) of caspase‐11 (green) and NRAS (magenta) staining in individual liver cells 6 days after hydrodynamic tail vein injection of NRAS with control‐ (a, b) or *Casp11*‐targeted (c, d) shRNA constructs. White arrows indicate NRAS + ve/caspase‐11 −ve cells (c). (e) qPCR data showing the level of *Casp11 *expression in livers 3 days after injection of constructs as indicated. (f, g) Representative images (f) and quantification (g) of NRAS staining (brown) in livers after the time and injection of constructs as indicated. (h) Quantification of F4/80 staining in livers treated as indicated. (i) Quantification of number of Ki67/NRAS double +ve cells in livers 6 days after injection of constructs. Data represent mean ± *SEM* of *n* = 5 (b, d, e, h, i), or as indicated (g); *p* = *≤0.05, **≤0.01, ****≤0.0001; ns = not significant. Scale bars = 50 μm (a, c), 300µm (f)

To investigate whether caspase‐11 is required for the SASP in vivo*, *we used a mouse liver model of NRAS‐induced senescence. Using hydrodynamic tail vein injection, we delivered *Nras*‐IRES‐*Casp11 *shRNA or *Nras*‐IRES‐Control shRNA constructs that undergo transposon‐mediated stable integration into hepatocytes (Kang et al., 2011). This system was chosen as it decreases caspase‐11 in only senescent cells, as opposed to all cells in *Casp11^−/−^*mice. Caspase‐11 protein was increased in NRAS‐positive cells after control shRNA treatment (Figure 5a), with ~97% of cells co‐staining for NRAS and caspase‐11 (Figure 5b), while reduced caspase‐11 protein (Figure 5c,d) and transcript (Figure 5e) was seen with the *Casp11*‐targeted construct. This indicates that caspase‐11 is upregulated during NRAS‐induced hepatocyte senescence in vivo and that *Casp11*‐targeted shRNA reduces expression. No difference in the number of NRAS‐positive cells between control‐ or *Casp11*‐targeted shRNA was seen at day 3, indicating equivalent integration and subsequent induction of senescence by NRAS (Figure 5f,g). However, *Casp11 *knockdown caused a significant accumulation of NRAS‐positive cells at days 6 and 12 (Figure 5f,g), and a significant negative correlation existed between the level of caspase‐11 and number of NRAS‐positive cells within livers (Supporting information Figure S10), indicative of a reduction in SASP‐driven immune‐mediated clearance. Consistent with this, the number of early infiltrating macrophages was decreased with *Casp11 *knockdown (Figure 5h), along with the number of immune cells clustering around NRAS‐positive cells (Supporting information Figure S11a,b). Importantly, the number of Ki67/NRAS double positive cells was equivalent between groups (Figure 5i), eliminating the possibility that accumulation of NRAS‐positive cells was due to *Casp11 *knockdown causing bypass of senescence. Together, this suggests that establishment of the IL‐1α‐dependent SASP that drives immune surveillance and clearance is dependent on caspase‐11 in vivo.

## 3 DISCUSSION

Due to the powerful, pleiotropic effects of IL‐1, unprecedented levels of control and feedback pathways are required to mount an immune response that resolves an insult without causing overt tissue damage. For example, multiple inflammasomes have evolved to distinguish tissue damage, environmental pollutants, bacteria, viruses, fungi and protozoa. IL‐1 also drives homeostatic processes such as thermoregulation (Dinarello, 2009), regulatory B‐cell differentiation (Rosser et al., 2014), intestinal integrity (Jung et al., 2015), and the immune surveillance and clearance of senescence cells (Kang et al., 2011). However, how IL‐1α is cleaved, activated and released was unknown.

We find that IL‐1α is directly cleaved by caspase‐5 and caspase‐11 at a conserved site, leading to full cytokine activity. Expression of a caspase site mutant IL‐1α or knockdown of *CASP5 *prevents release of cleaved IL‐1α from cells, while IL‐1α cleavage and release from murine macrophages require only caspase‐11, with IL‐1β needing both caspase‐11 and caspase‐1. Importantly, the IL‐1α‐dependent SASP requires caspase‐5 and caspase‐11 in vitro and in vivo. Thus, IL‐1α is a direct substrate of inflammatory caspases during noncanonical inflammasome activation and senescence.

The original *Casp1^−/−^*mice (Kuida et al., 1995; Li et al., 1995) showed loss of IL‐1β release from macrophages and during endotoxaemia. Unexpected reduced levels of IL‐1α were also found, which has remained a puzzle as IL‐1α is not a caspase‐1 substrate. However, *Casp1^−/−^*mice inherited a *Casp11 *inactivating passenger mutation from the 129/Sv background (Kayagaki et al., 2011). Our data suggest that defective release of cleaved IL‐1α in *Casp1^−/−^*mice is likely due to the absence of caspase‐11. Indeed, serum levels of cleaved IL‐1α are undetectable during endotoxaemia (which activates noncanonical inflammasomes via caspase‐11) in *Casp11^−/−^*and *Casp1^−/−^*/*Casp11^−/−^*mice, yet restored in the *Casp1^−/−^*/*Casp11* ^Tg ^(Kayagaki et al., 2011), supporting this concept. The primary role of caspase‐4/5 and caspase‐11 is thought to be LPS binding, and few substrates known. Gasdermin D is shown to be a key substrate for caspase‐11, caspase‐4/5 and indeed caspase‐1 (Kayagaki et al., 2015; Shi et al., 2015). We now report IL‐1α as a direct substrate for caspase‐5 and caspase‐11.

Although independent publications show cleavage of IL‐1α greatly increases activity (Afonina et al., 2011; Zheng et al., 2013), others contest this (Kim et al., 2013). Kim *et al *purified pro‐IL‐1α by HPLC, which typically denatures proteins due to the organic solvent mobile phase, and we find denatured and refolded pro‐IL‐1α has more activity than native pro‐IL‐1α (Zheng et al., 2013). Regardless, how IL‐1α is released and if cleavage is required was unknown. Gasdermin D‐mediated pore formation can allow IL‐1α to exit cells (Kayagaki et al., 2015; Shi et al., 2015). However, because all tested IL‐1α ELISA kits only recognize the cleaved form (K. Wiggins, M. Humphry, M. Clarke *unpublished data*), detection of IL‐1α within conditioned media represents both release and cleavage. Thus, active caspase‐5 or caspase‐11 could drive both the direct processing of IL‐1α and release via gasdermin D pores. In addition, pro‐IL‐1α is bound to IL‐1R2 inside many cells, which prevents activation by calpain (Burzynski et al., 2015; Zheng et al., 2013). However, caspase‐1 can cleave IL‐1R2 to enable IL‐1α activation, and this action may partly control canonical IL‐1α release (Zheng et al., 2013). Interestingly, caspase‐5, but not caspase‐4, also cleaves IL‐1R2 (Zheng et al., 2013). After canonical stimuli (e.g., LPS/ATP), IL‐1α release is dependent on ASC and NLRP3 (Kayagaki et al., 2011), while noncanonical stimuli only require caspase‐11 for cleaved IL‐1α release (Figure 3i and Kayagaki et al., 2011). This suggests that canonical IL‐1α release could proceed via NLRP3 caspase‐1 activation and cleavage of IL‐1R2, followed by calpain cleavage of IL‐1α, while noncanonical pathways could lead to IL‐1α release after direct cleavage of IL‐1R2 and IL‐1α by caspase‐5/11.

In murine systems, caspase‐11 binds LPS, increases caspase‐1 cleavage of IL‐1β (Wang et al., 1998), activates gasdermin D (Kayagaki et al., 2015; Shi et al., 2015) and now also directly cleaves and activates IL‐1α. In humans, an ancestral gene duplication resulted in *CASP4 *and *CASP5*, with caspase‐4 suggested as the closer homologue of caspase‐11 (Shi et al., 2014). Both caspase‐4 and caspase‐5 bind LPS and target gasdermin D, but only caspase‐5 can complement *Casp11^−/−^*cells (Shi et al., 2014), only *CASP5 *and *Casp11 *are upregulated in response to LPS (Figure 3a,b), and only caspase‐5/11 can cleave IL‐1α, suggesting a closer functional connection. We suggest caspase‐4 and caspase‐5 have most likely undergone subfunctionalization, with functionality of the ancestral *Casp11*‐like gene now distributed between both. However, if each performs nonredundant roles, and what these are, is still unknown.

Although IL‐1α induces sterile inflammation upon release from damaged cells, it also drives the SASP, but in this context IL‐1α release or surface presentation occurs without cell death (Figure 4g and Gardner et al., 2015). This excludes the usual notion that IL‐1α is passively released (Kayagaki et al., 2011). IL‐1α expression during the SASP is controlled in part by ATM/ATR liberation of GATA4 from p62‐directed autophagy (Kang et al., 2015) and/or an mTORC1‐dependent pathway (Laberge et al., 2015). Also, redox balance and Ca^2+ ^levels may enhance calpain processing of IL‐1α in senescent cells (McCarthy, Clark, Bartling, Trebak, & Melendez, 2013). Thus, how active IL‐1α is released without cell death during senescence was unknown. Here, we report increased *CASP5 *or *Casp11 *expression in senescent cells and a loss of cell surface and released IL‐1α without caspase‐5, leading to inhibition of the SASP. Interestingly, the SASP is not dependent on IL‐1β (Gardner et al., 2015; Orjalo et al., 2009), implying cleaved IL‐1α release occurs separately from IL‐1β (e.g., without caspase‐1/NLRP3/ASC), in contrast to other studies (Acosta et al., 2013). Indeed, IL‐1β release after icLPS requires caspase‐1 and caspase‐11, while IL‐1α only requires caspase‐11 (Figure 3g–j). Together, this suggests that cleavage of IL‐1α by caspase‐5/11 during senescence leads to release of active IL‐1α only, which drives the SASP. Interestingly, *CASP5 *mutations are associated with cancers (Offman et al., 2005), while *CASP1 *and *CASP4 *mutations are not (Soung et al., 2008), suggesting a caspase‐5‐specific protective role against tumorigenesis that is in keeping with SASP‐driven senescence surveillance.

In conclusion, we show that IL‐1α is a direct substrate of the noncanonical inflammatory caspase‐5 and caspase‐11, with cleavage increasing cytokine activity and release. Conservation of the cleavage site between species implies that activation of IL‐1α by caspase‐5 or caspase‐11 is important. Indeed, cleavage of IL‐1α by caspase‐5 or caspase‐11 is essential for the SASP. Thus, directly targeting caspase‐5 may reduce inflammation and limit the deleterious effects of senescent cells that accumulate during disease and ageing.

## 4 MATERIALS AND METHODS

All material from Sigma‐Aldrich unless otherwise stated.

### 4.1 Cell culture

HeLa cells (ECACC) and primary mouse fibroblasts were cultured in DMEM with penicillin, streptomycin, L‐glutamine and 10% FCS and passaged at 80% confluency. IMR‐90 and WI‐38 cells (ATCC) were cultured in phenol red‐free DMEM supplemented with penicillin, streptomycin and 10% FCS. Bone marrow‐derived macrophages (BMDMs) from wild‐type, *Casp11^−/−^*or *Casp1^−/−^Casp11* ^Tg ^(Kayagaki et al., 2011) mice (C57BL6/J background, 8–12 weeks old) were differentiated in RPMI 1640 with penicillin, streptomycin, L‐glutamine, 10% FCS and 15% L929 conditioned media, while immortalized BMDMs used the same media, but without L929. Human monocyte‐derived macrophages were isolated from whole blood (obtained with informed consent; NRES Committee, East of England) with Percoll gradients and differentiated in Iscove's with streptomycin, L‐glutamine and 10% autologous serum. IMR‐90 and WI‐38 cells were transduced with retrovirus carrying ER:*HRAS* ^G12V ^in pLNCX2 (Clontech), as previously described (Young et al., 2009), and senescence induced with 4‐hydroxytamoxifen (100 nM). All cells were checked for mycoplasma contamination (MycoAlert, Lonza). HeLa cells were transfected with pcDNA3 (Invitrogen) using FuGENE HD (Promega), while immortalized mBMDMs used Amaxa nucleofection (Lonza), before incubation (48h, 37°C). Knockdown in primary macrophages was performed with 10 pmol of nontargeting or *CASP5*‐targeting siGENOME siRNA pool (Dharmacon), while knockdown in IMR‐90 and WI‐38 cells was performed with 10 pmol of pooled or individual *CASP5‐ or cGAS‐*targeting siGENOME siRNAs, using Lipofectamine RNAiMAX (Invitrogen) as per the manufacturer's instructions. Cell viability was assessed by LDH release (Pierce), or crystal violet staining as previously described (Gardner et al., 2015).

### 4.2 IL‐1 Bioassays and inflammasome activation

HeLa cells or mouse fibroblasts were plated (10,000 cells/well, 48‐well plate) and incubated (16 hr, 37°C). Media was refreshed and cells incubated (6 hr, 37°C) ± cleavage assay products (1.5 µl). Specific IL‐1 activity was inferred by the effect of neutralizing antibodies (2 µg/ml; same as for Westerns), on IL‐6 production. Every experiment contained a media only negative control and a recombinant IL‐1 (10 ng/ml; PeproTech) positive control. For noncanonical, cells were primed with ultrapure LPS (1 µg/ml, 4 hr; InvivoGen) in full media, transfected with ultrapure LPS (5 µg/ml) using FuGENE HD (2.5 µl/ml; Promega) and incubated in Opti‐MEM (37˚C, 18 hr). For canonical, cells were primed with ultrapure LPS (1 µg/ml, 4 hr) in full media, treated with ATP (5 mM, pH7; InvivoGen) and incubated in Opti‐MEM (37˚C, 18 hr).

### 4.3 Recombinant protein expression, purification and cleavage

Pro‐IL‐1α/β or pro‐caspase‐5 cDNAs were cloned into pGEX‐4T‐3 (GE) or pcDNA3 (Invitrogen), with mutations introduced by site‐directed mutagenesis. For bacterial expression, IPTG‐induced Rosetta cultures were lysed (PBS 1×; DTT 1 mM; EDTA 10 mM; benzonase 30 U/ml; lysozyme 55 kU/g (both Novagen); protease inhibitor cocktail) with sonication, and clarified by centrifugation (5,525 g, 1 hr, 4°C). Filtered supernatants were loaded onto a GSTrap column (GE), washed (PBS; DTT 1 mM; EDTA 1 mM) and eluted (Tris 50 mM; NaCl 100 mM; DTT 1 mM; reduced l‐glutathione 50 mM; adjusted to pH 8) using an ÅKTA FPLC system (GE). Eluted protein was dialysed overnight (Tris 10 mM, pH8; NaCl 50 mM) and stored in glycerol (10%) at −80°C. IL‐1 protein concentration was normalized by SDS‐PAGE, Coomassie staining (G‐250; Bio‐Rad) and quantitative imaging (Odyssey, Li‐Cor). Recombinant IL‐1 (4 µg/ml) was incubated (1 hr, 37°C) with active caspase (100 U or 10 U; all Enzo) in reaction buffer (human—HEPES 50 mM, pH 7.4; NaCl 100 mM; CHAPS 0.1%; EDTA 1 mM; glycerol 10%; DTT 10 mM) (mouse—MES 100 mM, pH 6.5; CHAPS 0.1%; PEG 10%; DTT 10 mM). Where indicated, LEVD‐FMK (330 µM; Enzo) was added. Recombinant IL‐1 (4 µg/ml) was incubated (1 hr, RT) with active calpain (100 U; Calbiochem) in reaction buffer (NaCl 100 mM; CaCl2 2 mM; DTT 1 mM).

### 4.4 Induction of mouse hepatocyte senescence in vivo

Mice were handled and kept under pathogen‐free conditions in accordance with UK law and institutional guidelines at the University of Cambridge and CRUK Cambridge Institute. Transposon‐mediated gene transfer by hydrodynamic tail vein injection was as previously described (Dauch et al., 2016; Kang et al., 2011). Briefly, short‐hairpin oligomers targeting *Casp11 *(5’‐TACCATCTATCAGATATTCAA‐3’) were cloned into MSCV‐miR30‐puro before subcloning into pCaNIG‐miR30 (from Lars Zender (Dauch et al., 2016)). Vectors were prepared using the EndoFree MaxiPrep Kit (Qiagen). Female C57BL/6 mice (Charles River) were injected via the lateral tail vein with pCaNIG‐miR30 (20 µg) and SB13 transposase vector (5 µg) in PBS at a volume equivalent to 10% of body weight in <10 s, and livers harvested at the times indicated.

### 4.5 Western blotting and cytokine detection

Western blotting was performed as previously described. Blocked PVDF membranes (Bio‐Rad) were incubated (16 hr, 4°C) with anti‐human IL‐1α (500‐P21A, PeproTech; 1:500), anti‐mouse IL‐1α (AF‐400NA, R&D; 1:500), anti‐human IL‐1β (AF‐201‐NA, R&D; 1:500) or anti‐mouse IL‐1β (500‐P51, PeproTech; 1:500). Binding was detected with: anti‐rabbit HRP (NA934V, GE) or anti‐goat HRP (805‐035‐180, Jackson ImmunoResearch) (both 1:2000), ECL reagents (GE) and apposition to X‐ray film (Fujifilm). Cytokines were measured using human or mouse IL‐1α, IL‐1β, IL‐6, IL‐8 and MCP‐1 bead immunoassays (Life Technologies), as per the manufacturer's instructions, and assayed with a flow cytometer (Accuri C6). The antibody binding to the region between the calpain and caspase‐5 cleavage site in IL‐1α was generated by immunizing rabbits with a KLH‐conjugated peptide, followed by affinity purification and biotinylation (Innovagen). This was then used as the detection reagent in a standard sandwich ELISA using a goat anti‐human IL‐1α antibody (R&D) as capture.

### 4.6 Gene expression analysis

RNA was isolated (RNeasy, Qiagen) and converted to cDNA using AMV reverse transcriptase (Promega). qPCR used TaqMan probes with AmpliTaq Gold (Life Technologies) in a Rotor‐Gene thermocycler (Corbett). Gene expression was evaluated using the 2^−ΔΔCT^ method using GUSB as a reference gene. qPCR data are displayed as 2^−ΔΔCT^, but statistical analysis was performed on the untransformed ΔΔCT values. RNA‐Seq data were from GEO GSE72404.

### 4.7 Assessment of Cell Surface IL‐1α

Cells were detached (Accutase), fixed (2% formaldehyde; 5 min, RT), washed (BSA, 0.5%; NaN3, 0.05%; in PBS), Fc blocked (10 min, RT; BioLegend; 1:100), before incubation (30 min, RT) with anti‐IL‐1α‐FITC (FAB200F, R&D; 1:20) or isotype control‐FITC (IC002F, R&D; 1:20), before washing and analysis by flow cytometry (Accuri C6).

### 4.8 SAβG, BrdU and SAHF staining

For SAβG staining, cells were washed (PBS), fixed (0.5% glutaraldehyde, 10 min, RT), washed (MgCl2 1 mM/PBS) and stained in X‐gal solution (X‐gal, 1 mg/ml; K3Fe[CN]6, 0.12 mM; K4Fe[CN]6, 0.12 mM; MgCl2, 1 mM; in PBS, pH 6.0) overnight (37°C). For BrdU and SAHF with 5‐bromo‐2’‐deoxyuridine (BrdU; 100 µg/ml), fixed (4% formaldehyde; 15 min, RT), permeabilized (0.2% Triton X‐100/PBS), blocked (0.5% goat serum) and incubated with anti‐BrdU (555,627, BD; 1:500), washed (Tween 0.05%/PBS; 5 min) and incubated (1 hr) with goat anti‐mouse Alexa 488 (A‐11034, Thermo Fisher; 1:500). Cells were mounted in VECTASHIELD (Vector Laboratories) with DAPI (1 µg/ml) and imaged with a TCS SP8 microscope (Leica).

### 4.9 Immunofluorescence and Immunohistochemistry

After processing, paraffin sections were cleared before antigen retrieval with sodium citrate (10 mM; pH 6), blocking with M.O.M. (Vector Laboratories) and donkey serum (0.5%, 1 hr), and then incubated (4°C, 16 hr) with antibodies to anti‐NRAS (sc‐31, Santa Cruz; 1:250), anti‐caspase‐11 (MAB86481, R&D; 1:50), anti‐F4/80 (MCA497, Serotec; 1:100) and anti‐Ki67 (GTX16667, GeneTex; 1:500). After washing (Tween 0.05%/PBS; 5 min), primary antibodies were visualized with donkey anti‐rat Alexa 488 (ab150153, Abcam), donkey anti‐mouse Alexa 647 (A31571, Invitrogen), donkey anti‐rabbit Alexa 555 (A31572, Invitrogen; all 1:250) or the EnVision + kit (K500711‐2, Agilent Technologies).

Autofluorescence was reduced by incubation (10 min, RT) with Sudan Black (0.1% in 70% EtOH) before mounting in VECTASHIELD (Vector Laboratories) with DAPI (1 µg/ml), or counterstained with haematoxylin. Slides were imaged or scanned on an Aperio AT2 (Leica). Images were analysed with HALO (Indica Labs) and the Cytonuclear v1.4 algorithm.

### 4.10 Statistical Analysis

All statistical analyses were carried out using Prism 7 (GraphPad). Data were analysed by unpaired *t* test (two‐tailed), one‐way ANOVA with Dunnett's post hoc or one‐way ANOVA with Tukey's post hoc. *n* = individual biological replicate performed in duplicate. The in vitro experiments typically required sample sizes of *n* = 3–4 to provide adequate power, and produced data that were normally distributed.

## CONFLICT OF INTEREST

None declared.

## AUTHOR CONTRIBUTIONS

KW, AP, LC, SW and MH designed and performed experiments, and analysed data. JG and MN designed experiments, analysed data and provided helpful discussions. MC conceived the project, designed experiments, analysed data and wrote the manuscript with KW.

## DATA AVAILABILITY

The data that support the findings of this study are available from the corresponding author upon reasonable request.
