# Erratum

**DOI:** 10.1111/acel.13082

**Published:** 2019-12-05

Wiggins, KA, Parry, AJ, Cassidy, LD, et al. IL‐1α cleavage by inflammatory caspases of the noncanonical inflammasome controls the senescence‐associated secretory phenotype. Aging Cell. 2019; 18:e12946. https://doi.org/10.1111/acel.12946

In the article “IL‐1α cleavage by inflammatory caspases of the noncanonical inflammasome controls the senescence‐associated secretory phenotype,” a duplication of the image in Figure 1 inadvertently happened during the production process. The correct Figure 1 is shown below.

**Figure 1:**
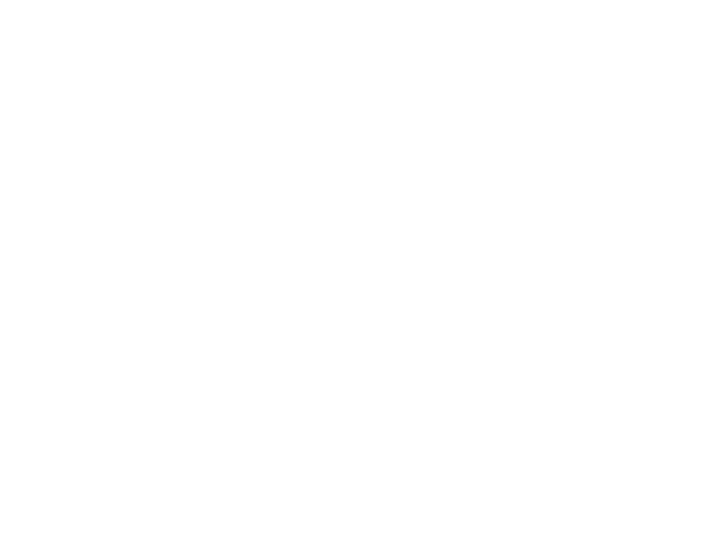
Caspase‐5 cleavage of human IL‐1α at a conserved site increases activity. (a) Western blot for IL‐1α after incubation of pro‐IL‐1α with active caspases, or alone (incubation control; IC). (b) IL‐1‐dependent IL‐6 production by HeLa cells treated with reaction products from pro‐IL‐1α incubated ±active caspases, ±neutralizing IL‐1α antibody (α pAb). (c, d) Western blot (c) and bioactivity (d) of IL‐1α after incubation ±caspase‐5, ±caspase inhibitor LEVD. (e) Multispecies IL‐1α protein alignment showing conserved aspartic acid residue (arrow). (f) Western blot for wild‐type (WT) or mutant D^103^A pro‐IL‐1α after incubation ±caspase‐5, or alone (IC). (g) IL‐1‐dependent IL‐6 production by HeLa cells treated with reaction products from WT or mutant pro‐IL‐1α incubated ±caspase‐5, ±neutralizing IL‐1α antibody (α pAb). (h) Pictograph showing position of cleavage sites in IL‐1α. Data represent mean ± *SEM*. of *n* = 3 (g), *n* = 4 (b, d); *p* = **≤0.01, ***≤0.001, ****≤0.0001; ns = not significant

We apologize for the inconvenience caused.

