# Supplementary material for: IL‐1α cleavage by inflammatory caspases of the noncanonical inflammasome controls the senescence‐associated secretory phenotype
Source: Aging Cell. 2019 Mar 27;18(3):e12946. doi: 10.1111/acel.12946 (PMC6516163; doi:10.1111/acel.12946)
Supplement: Supplementary file 1 [file ACEL-18-e12946-s001.docx]

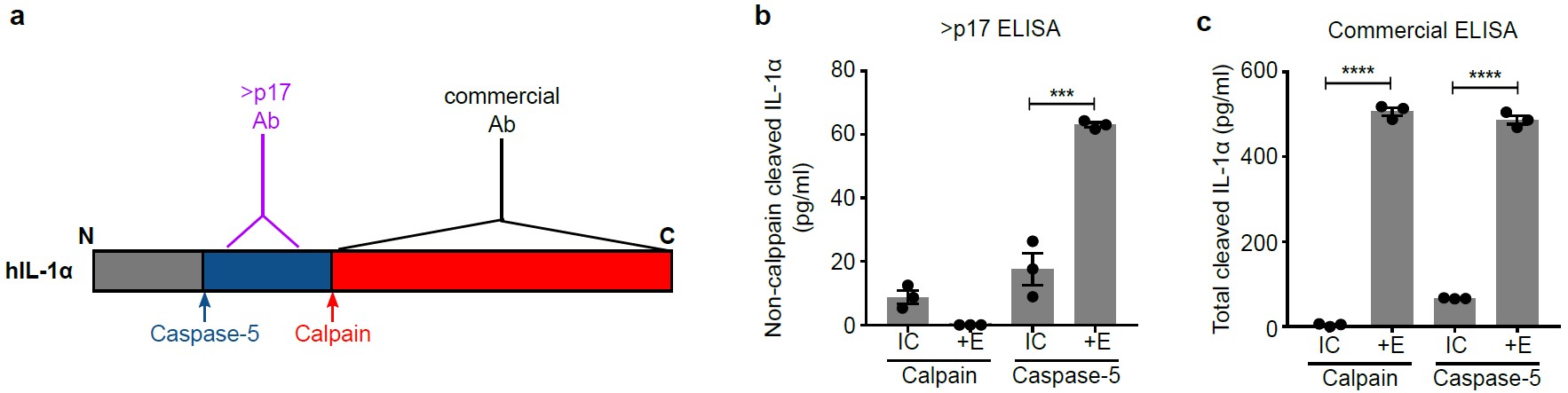


**Supplementary Figure 1: (a)** Schematic showing location of the custom peptide antibody relative to the calpain and caspase-5 cleavage sites in pro-IL-1α. **(b-c)** Data showing specificity of our custom non-calpain cleaved IL-1α ELISA **(b)** or a total cleaved IL-1α ELISA **(c)** for detecting either calpain or caspase-5 cleaved pro-IL-1α. IC = incubation control (no enzyme); +E = +enzyme. Data represent mean ± s.e.m. of n = 3; p= ***≤0.001, ****≤0.0001.


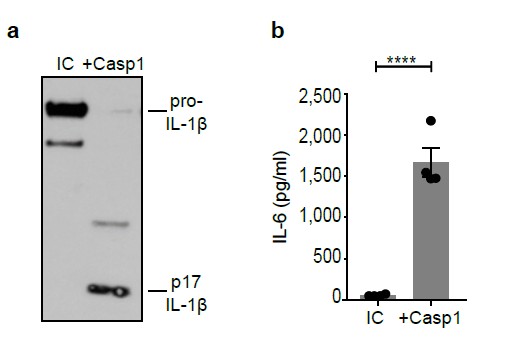


**Supplementary Figure 2: (a)** Western blot for IL-1β after incubation of murine pro-IL-1β with murine caspase-1, or alone (IC). **(b)** IL-1- dependent IL-6 production by murine fibroblasts treated with reaction products from murine pro-IL-1β incubated ± murine caspase-1. Data represent mean ± s.e.m. of n = 4; p= ****≤0.0001.

**a b**


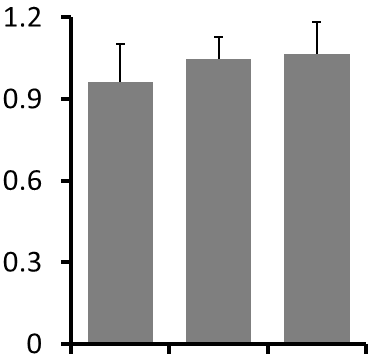


ns

- *Il1aWT*

*Il1a106A*


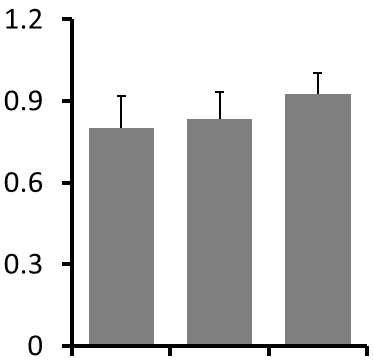


ns

- *Il1aWT*

*Il1a106A*

LDH (+icLPS/-icLPS)

Cell # (+icLPS/-icLPS)

pcDNA3

pcDNA3

**Supplementary Figure 3: (a,b)** Analysis of cell viability by release of lactate dehydrogenase (LDH) into the conditioned media **(a)** and number of cells attached by crystal violet staining **(b)** in immortalised mBMDMs after transfection of empty, WT *Il1a* or D106A *Il1a* vectors, followed by LPS priming and then transfection of intracellular LPS (icLPS). Data represent mean ± s.d. of n = 3; ns = not significant.

Senescent


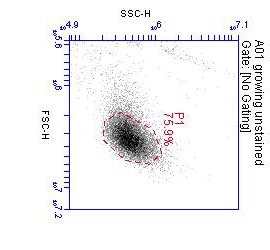

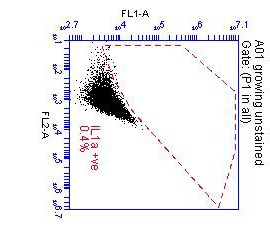

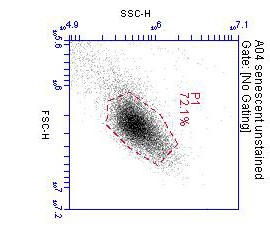

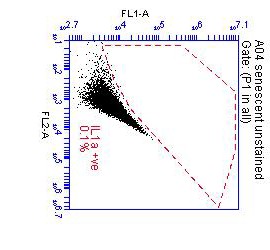

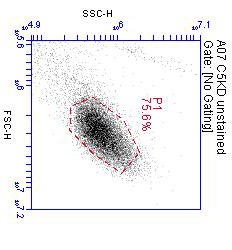

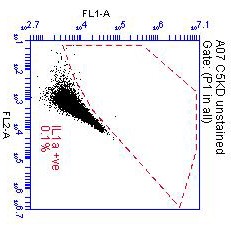

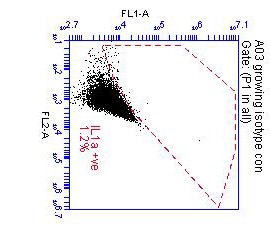

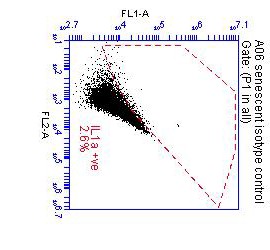

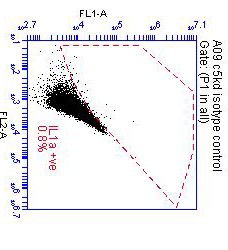

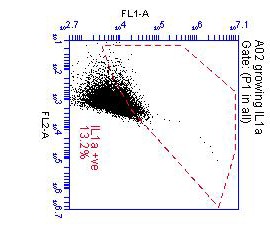

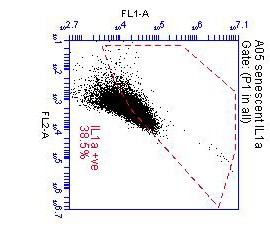

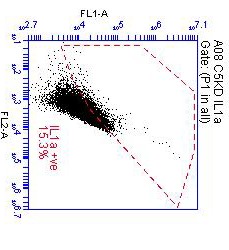


Unstained

Isotype-FITC

+*CASP5* KD Senescent Growing

FSC/SSC

**Supplementary Figure 4:** Representative flow cytometry dot plots of growing IMR-90 cells, senescent IMR-90 cells and senescent IMR-90 cells after CASP5 knockdown, which were left unstained or stained with isotype control-FITC or anti-IL-1α-FITC antibodies before analysis by flow cytometry.

IL-1α-FITC


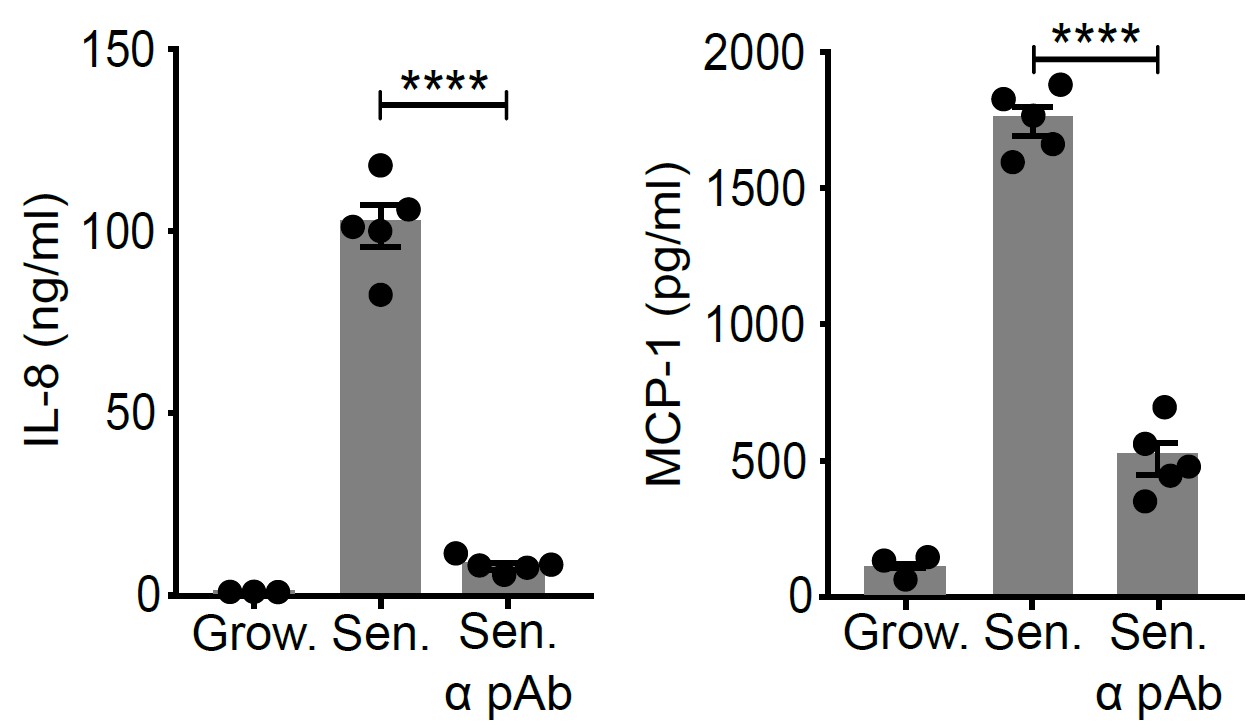


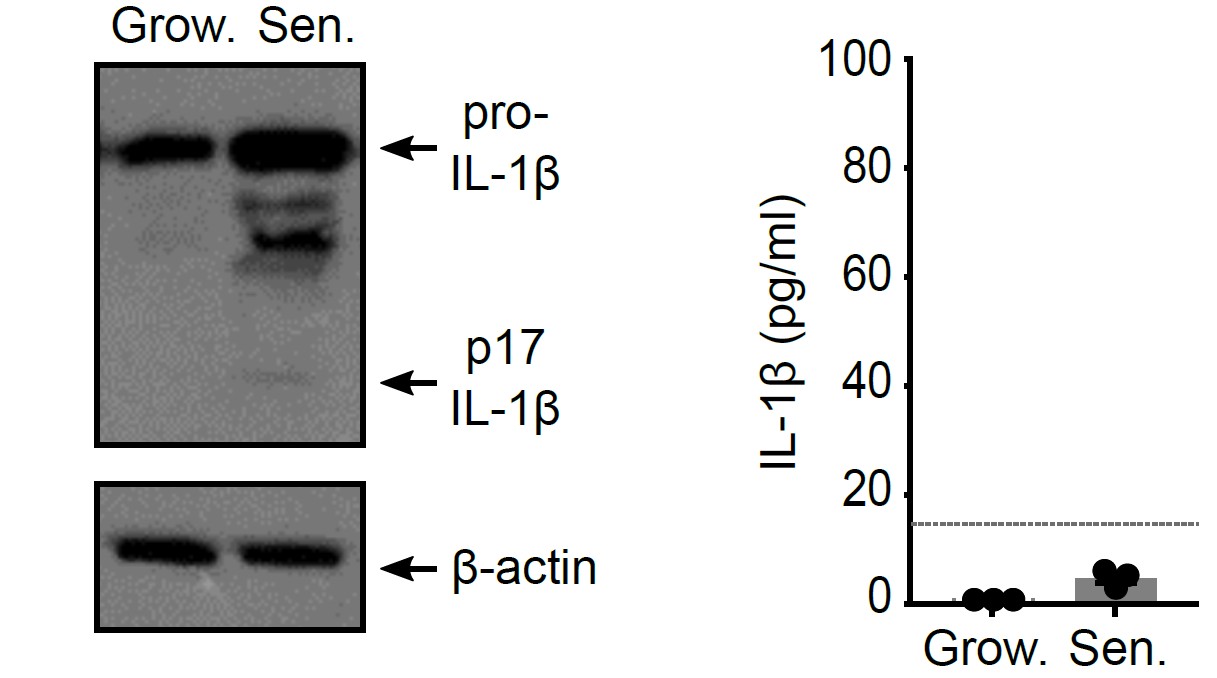


**a**

**b**

**Supplementary Figure 5:** ELISA data showing the level of IL-8 and MCP-1 in the conditioned media of growing (Grow.) or senescent (Sen.) IMR-90 cells, ± neutralising IL-1α antibody (α pAb). Data represent mean ± s.e.m. of n = ≥3; p = ****≤0.0001.

**Supplementary Figure 6: (a)** Western blot for IL-1β in growing (Grow.) and senescent (Sen.) IMR-90 cells. **(b)** ELISA data showing the level of IL-1β in the conditioned media of growing and senescent IMR-90 cells. Grey dotted line indicates limit of detection. Data represent mean ± s.e.m. of n = 3.

**a** Growing


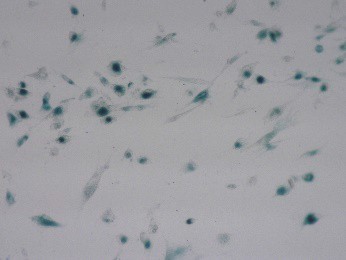


Senescent


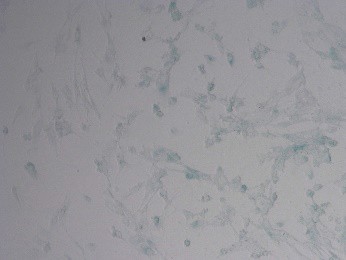


SAβG


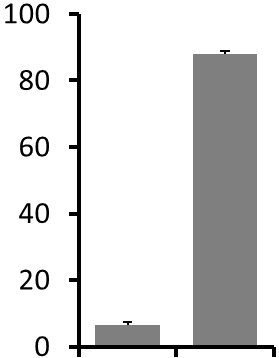


****

WI-38 cells

SAβG positive (%)

**b
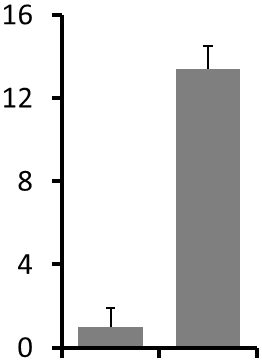
 d** **


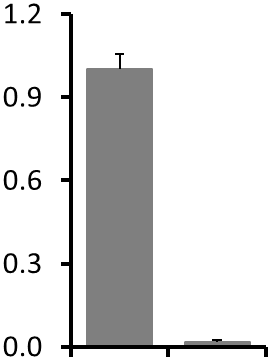


*CASP5* Expression

Cleaved IL-1α (pg/ml)

IL-6 (ng/ml)

IL-8 (ng/ml)

| Grow. Sen. | Grow. Sen. | Grow. | Sen. | Sen. Sen. | Sen. | Sen. |
| --- | --- | --- | --- | --- | --- | --- |
|  |  |  |  | α pAb |  | α pAb |


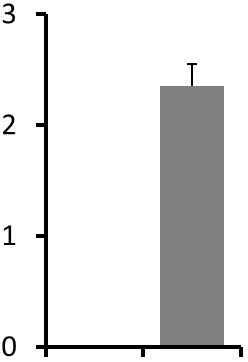

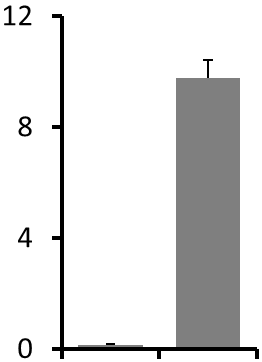
 **c**

IL-6 (ng/ml)

IL-8 (ng/ml)

**e** **** *


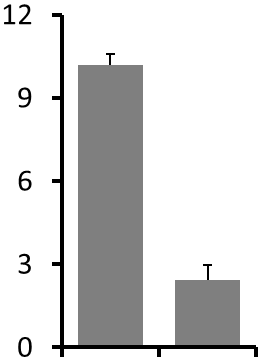

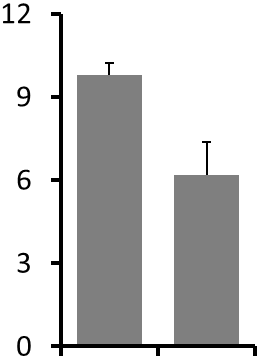


Grow. Sen.


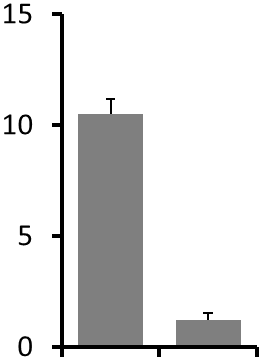


***


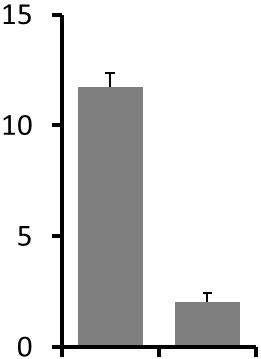


***

IL-6 (ng/ml)

IL-8 (ng/ml)

Ctrl *CASP5*

siRNA

Ctrl *CASP5*

siRNA

Ctrl *CASP5*

siRNA

**Supplementary Figure 7: (a)** Representative images and quantification of senescence-associated beta galactosidase (SAβG) staining in growing (Grow.) and senescent (Sen.) WI-38 cells. **(b,c)** ELISA data showing the level of cleaved IL-1α and/or SASP factors in the conditioned media of growing and senescent WI-38 cells **(b)**, or senescent WI-38 cells ± neutralising IL-1α antibody (α pAb) **(c)**. **(d,e**) Relative *CASP5* expression by qPCR **(d)** or SASP factors in the conditioned media by ELISA **(e)** in senescent WI-38 cells after transfection of control (Ctrl) or *CASP5*-targeted siRNA. Data represent mean ± s.e.m. of n = ≥3 **(a,c-e)**; or mean ± s.d. of n=2 **(b)**; p =

*≤0.05, **≤ 0.01, ***≤0.001, ****≤0.0001.


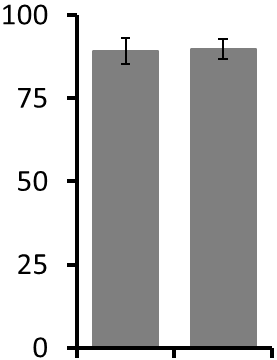
**a b**


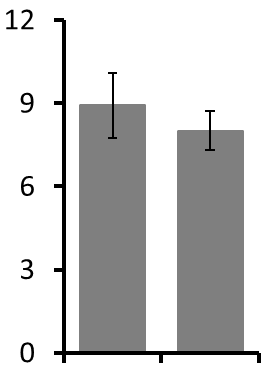


Ctrl *CASP5*


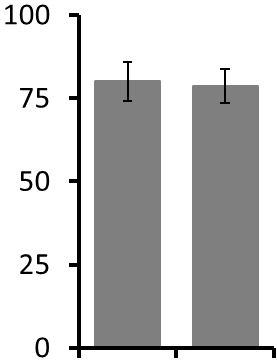


Ctrl *CASP5*


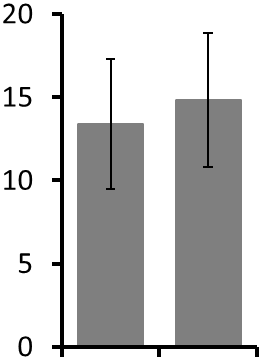


Ctrl *CASP5*

SAβG +ve cells (%)

BrdU +ve cells (%)

SAβG +ve cells (%)

BrdU +ve cells (%)

siRNA

siRNA

Ctrl *CASP5*

siRNA

siRNA

**Supplementary Figure 8: (a,b)** Level of SAβG and BrdU +ve cells in senescent IMR-90 **(a)** and WI-38 **(b)** cell cultures after transfection

of control (Ctrl) or *CASP5*-targeted siRNAs. Data represent mean ± s.d. representative of n = 2. 17


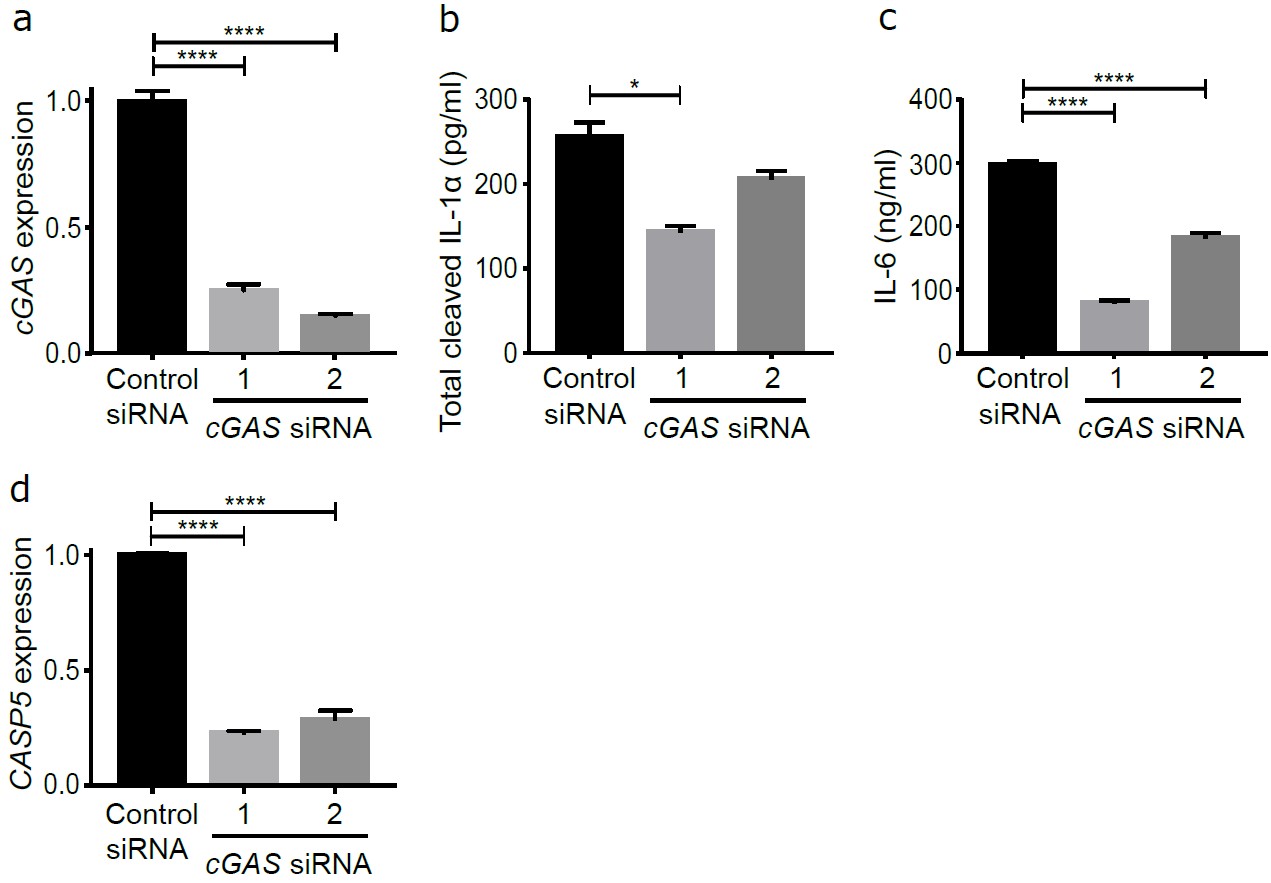


**Supplementary Figure 9: (a)** qPCR data showing relative expression of cGAS in senescent IMR-90 cells after transfection of control or *cGAS*-targeted siRNAs. **(b-d)** Total cleaved IL-1α **(b)** and IL-6 **(c)** by ELISA in the conditioned media, or *CASP5* expression by qPCR **(d)** in senescent IMR-90 cells after transfection of control or *cGAS*-targeted siRNAs. Data represent mean ± s.e.m. of n = 3 **(a-c)**, n = 6 **(d)**; p = *≤0.05, ****≤0.0001.


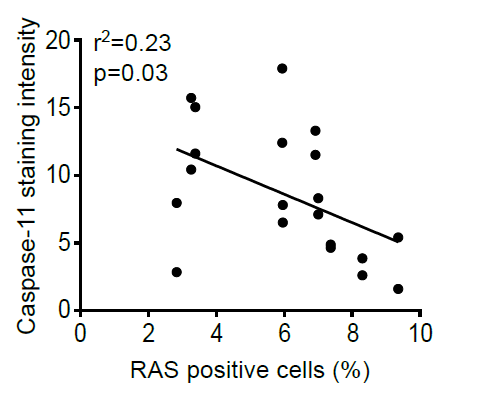


**Supplementary Figure 10:** Linear regression analysis of the correlation between average caspase-11 staining intensity and percentage of RAS positive cells in mouse livers 6 days after injection.

**a b**


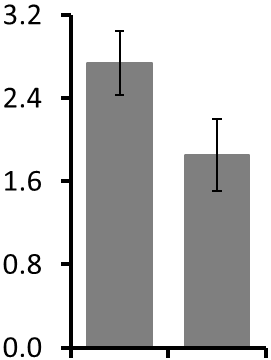


**


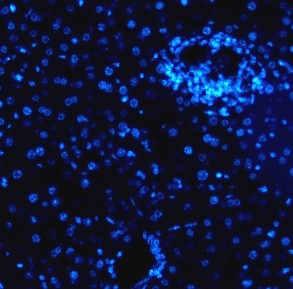


DAPI


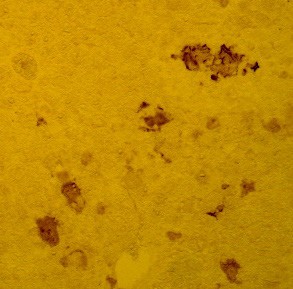


NRAS

Immune clusters / mm2

Ctrl *Casp11*

Mouse Liver

shRNA

**Supplementary Figure 11: (a)** Representative images of immune cells clustering around two NRAS+ senescent cells (arrows) within mouse liver 6 days after hydrodynamic tail vein injection of NRAS. **(b)** Quantification of immune cell clusters around NRAS+ cells within mouse liver 6 days after hydrodynamic tail vein injection of NRAS with control (Ctrl) or *Casp11*-targeted shRNA constructs. Data represent mean ± s.d. of n = 5 mice; p = **≤0.01.
